# Azathioprine: its uses in dermatology^⋆^^⋆⋆^

**DOI:** 10.1016/j.abd.2020.05.003

**Published:** 2020-09-11

**Authors:** Sonia Chavez-Alvarez, Maira Herz-Ruelas, Alejandra Villarreal-Martinez, Jorge Ocampo-Candiani, Rubicela Garza-Garza, Minerva Gomez-Flores

**Affiliations:** a Department of Dermatology, University Hospital “Dr. José Eleuterio González”, Universidad Autónoma de Nuevo León, Nuevo León, Mexico; b Private Dermatology Practice, San Pedro Garza Garcia, Nuevo Leon, Mexico

**Keywords:** Autoimmunity, Azathioprine, Pemphigus

## Abstract

This is a narrative review of azathioprine. This medication is immunomodulatory and immunosuppressive, and it has been used widely through different medical specialties to modify disease. It has been proven useful for several dermatoses and it has encountered success when used as an off-label indication for other dermatologic diseases. Its mechanism of action is described thoroughly, as well as precautions for monitoring adequate levels in patients using it. Dermatologists should also be aware of the possible adverse events it may present. In dermatology it can be used in bullous and autoimmune diseases, and in other conditions, including intractable pruritus, atopic dermatitis, photodermatoses, psoriasis, and others. Azathioprine offers an alternative as a steroid-sparing agent and this review helps dermatologists prescribe it safely to all patients who require it.

## Introduction

Azathioprine was first used to prevent graft-versus-host disease. Today it is widely used as an immunomodulator, immunosuppressant, and a steroid-sparing agent.

It is a pro-drug that is rapidly converted to 6-mercaptopurine (6-MP) by means of the purine metabolism pathway, and its therapeutic effects are derived from its purine anti-metabolism.^1^ As a purine analogue, it inhibits DNA production and exerts effects on cells with a high proliferation rate (*i.e*., T and B lymphocytes).^2^

It is an approved to prevent graft *vs*. host disease in organ transplant recipients, severe rheumatoid arthritis, systemic lupus erythematosus, and atopic dermatitis.^2^, ^3^ In dermatology, it is used off-label for other several conditions.^2^, ^3^

## Metabolism and pharmacodynamics

**Figure 1:**
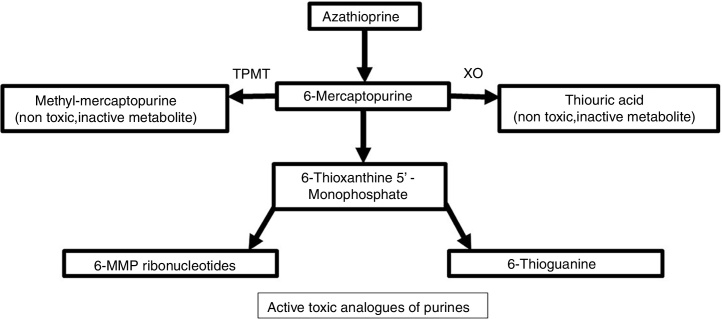
Azathioprine metabolism.

It is synthesized from 6-MP and an imidazolic ring in the sulfur atom which stabilizes the molecule and avoids immediate catabolism.^4^ This purine antimetabolite inhibits the S phase of the cell cycle. The metabolism of endogenous purines produces 6-MP.^5^ Three metabolic pathways exist within liver and erythrocytes: xanthine oxidase, thiopurine methyltransferase (TPMT), and hypoxanthine phosphoribosyltransferase. The latter is responsible for the drug's activity, while the first two are the main catabolic pathways and produce inactive metabolites like thiouric acid (Fig. 1).^5^, ^6^

For 6-MP to affect the synthesis of nucleic acids, it needs to be converted into thioinosinic acid.^7^ This nucleotide inhibits mitosis, coenzyme formation, neutrophils, and monocytes, as well as suppressing the synthesis of prostaglandins by means of cyclooxygenase (COX).^7^ It has a selective activity for T over B lymphocytes.^8^ Between 70% and 80% of the drug is absorbed within the gastrointestinal tract and it reaches peak serum levels two hours after ingestion.^7^, ^9^ It does not cross the blood-brain barrier.^4^

### Dose

Adults require 1–3 mg/kg/day and 1–4 mg/kg/day is the pediatric dose.^2^, ^5^ Therapeutic effects are seen one to two months after starting the drug. Dose adjustments must be made according to each patients’ response.^5^, ^8^ Administration with meals in divided doses is suggested.^3^ Maintenance can extend to 93 months.^1^

### Precautions before treatment

It is advisable to thoroughly explain information regarding this medication to the patient. Those unable to be closely monitored are not eligible for treatment.^3^

### Request:

1. Antigens for hepatitis B and C, enzyme-linked immunosorbent assay (ELISA) for HIV.
2. Human chorionic gonadotropin (if indicated).
3. Complete blood cell count, blood chemistry, and liver function tests, before treatment and twice a month for the first three months. Finalizing this period, a bimonthly follow-up is required.
4. Annual tuberculosis screening.^2^

Hepatic or renal malfunction mandates lower doses. If suspension of the medication is required, adverse effects will gradually diminish because of persistence of the active metabolite.^5^

Patients without a history of varicella zoster infection receiving treatment and recent close contact with the virus should be promptly treated with immunoglobulin. Administration of live virus vaccines for the patient and relatives is prohibited due to the existing risk of transmission. Family members should receive inactivated virus vaccines.^3^

Children over 6 months of age and those not responding to safer medications are eligible. Treatment for short periods may induce prolonged remissions. Dose tapering must be done in six months up to one year.^4^

Fertility is not adversely affected; however, there is scarce evidence.^4^ It is advisable to avoid pregnancy and use contraception during treatment.^3^ Adverse events include a preterm or underweight child.^10^, ^11^ Azathioprine passes the placental barrier and the fetal liver lacks enzymes required for its conversion into active metabolites.^9^

Hematological toxicity and sporadic anomalies are also described. There is no established pattern of congenital malformations (which may include atrial or ventricular septal defects). The malformation rate varies from 3% to 9% and includes myelomeningocele, microcephaly, preaxial polydactyly, thymic atrophy, and adrenal hypoplasia and/or hypospadias.^9^

It is a category D drug in pregnancy (acceptable benefits but risks to fetuses). Its use should be reserved for life threatening diseases.^12^ Patients should receive half the dose in the 32^nd^ week of gestation if their leukocyte count is less than one standard deviation below the mean. This helps prevent leukopenia and/or thrombocytopenia in the baby.^10^ A study involving pregnant women receiving azathioprine for eight weeks in the first trimester resulted in scarce evidence linking it to congenital malformations, low birth weight, and/or prematurity.^13^ In males, no anomalies in seminal fluid three months after initiation of treatment were described in patients with inflammatory bowel disease (IBD).^14^

Breastfeeding is not advisable during treatment due to the risk of tumorigenesis and increased infections in infants.^15^ However, a three-year follow-up study in children of patients with IBD receiving azathioprine found no significant differences in the rate of infections compared with children of healthy mothers.^16^ Most of the 6-MP is excreted in milk four hours following ingestion, though the quantity ingested by infants is not considered significant.^17^

### TPMT deficiency

TPMT is an inducible enzyme and its levels vary with time.^2^ Initial measurement of this enzyme is required. Otherwise, there is a risk of adverse effects or treatment at subtherapeutic doses.^2^ Myelosuppression (neutropenia and pancytopenia) may be present with regular doses. An unidentified pancytopenia may become lethal.^5^ Ten percent of individuals have low TPMT activity, which generates thiopurine toxicity.^5^ Resultant accumulation of thioguanine nucleotides affect bone marrow.^8^ Those with no TPMT activity should not be prescribed azathioprine.^5^ A greater activity of TPMT confers less risk to induce a myelosuppressive response (Table 1).^2^

**Table 1:** Thiopurine methyltransferase (TPMT) levels and azathioprine dosage

TPMT levels	Dosage of azathioprine
<5 U	Contraindicated
5–13.7 U	Up to 0.5 mg/kg/day
13.7–19 U	Up to 1.5 mg/kg/day
>19 U	Up to 2.5 mg/kg/day

Adapted from Patel et al.^4^

## Applications in dermatology

### Pemphigus vulgaris

Used as first-line steroid sparing agent. It offers better results as compared to mycophenolate mofetil (MMF).^5^, ^18^ Another drug, cyclophosphamide, has a faster onset of action; however, treatment effectiveness is comparable at six months.^19^ When combined with prednisone, final outcomes are better, with benefits such as a greater number of patients in remission and less adverse effects (Table 2).^20^

**Table 2:** Azathioprine contraindications.

Relative	Absolute
Use of allopurinol	6 MP hypersensitivity or azathioprine
Previous treatment with cyclophosphamide or chlorambucil	Severe active infection
Renal failure	Altered liver function
Viral hepatitis, HIV	Altered bone marrow function
Previous infection by VZV	Pancreatitis
Pre-malignancy	Live virus vaccines
	Pregnancy/breastfeeding

### Bullous pemphigoid

Starting a steroid-sparing agent since the initiation of treatment is advisable. Time of onset of the therapeutic effects for azathioprine varies from one week to seven months. At a four-year follow-up, 44% of patients achieve remission of the disease.^1^ It halts disease progression and promotes re-epithelialization as soon as eight weeks of starting treatment.^21^ MMF is less hepatotoxic, but five times as costly. A faster and complete cure was also demonstrated when compared with MMF (23.8 *vs*. 42 days).^22^ However, no differences in disease control have been identified with prednisolone alone, azathioprine and prednisolone, prednisolone with plasmapheresis, or prednisolone with azathioprine or MMF.^23^

### Chronic actinic dermatitis

It induces 57% to 92% of improvement in patients when compared with placebo. Dosage varies between 1–2.5 mg/kg. Adverse effects observed in patients treated with azathioprine for this condition include three deaths, 12–15 months after stopping treatment (secondary to cerebrovascular disease, lung disease, and another due to heart disease).^1^ It has demonstrated effectiveness in refractory disease. Drug interactions need to be reviewed since these patients have polypharmacy, which is why it is preferred over cyclosporine.^24^

### Psoriasis

It may be indicated for patients with refractory disease.^1^ It can be used in conjunction with biologicals (not recommended as monotherapy).^5^ Combined with infliximab (5 mg/kg), it induces improvement.^25^ Intermittent pulse monotherapy may be effective giving 500 mg doses administered for three consecutive days each month, and 100 mg daily continuously for 12 to 24 months. Remission may be achieved for more than five years in some patients.^26^

### Atopic dermatitis

Information is scarce; however, it has been shown to be as effective as methotrexate.^1^, ^27^, ^28^ It is recommended for recalcitrant variants in pediatric patients or when there is a significant psychosocial impact on the patient and their family.^2^

It can be used in combination and as a steroid-sparing agent.^1^ It reduces the SAS-SAD scale by 26% when compared with placebo (3%) after three months of treatment.^29^ When used for 12 weeks, the activity of the dermatosis was reduced by 37% *vs.* 20% in the placebo group.^30^ A 27% improvement in the severity scoring of atopic dermatitis (SSAD) was also demonstrated after six months.^1^

### Cutaneous vasculitis

It is effective when combined with prednisolone for purpura, ulcers, nail fold microinfarcts, and/or peripheral necrosis at 2 mg/kg. There is significant improvement in vasculitis after 18 months of treatment, and 88% of patients achieved complete remission. Side effects included septic arthritis, epidural abscess, gastrointestinal effects, and an isolated death from renal failure (due to severe vasculitis).^1^

For leukocytoclastic vasculitis it is recommended as a second-line therapy in steroid resistant Henoch–Schoenlein purpura, but it has been used successfully and has improved the course of nephritis.^31^

In ANCA vasculitis, azathioprine *vs.* rituximab were compared to maintain remission of the disease. More patients remained in remission at 28 months with rituximab *vs*. azathioprine.^32^

### Intractable pruritus

In a retrospective study of patients with chronic pruritus (85% of patients with symptoms for 12 months or more) who transiently responded to a course of systemic steroids but were refractory to other treatments, azathioprine was initiated, with a mean starting dose of 137.5 mg/day. The pruritus scale was modified from a 9 to a 1 or 2.^33^

### Connective tissue diseases (lupus)

Useful in refractory subacute cutaneous lupus erythematosus, generalized discoid lupus, and the erosive palmoplantar type, demonstrating a complete or partial improvement. Rowell's syndrome shows good therapeutic response when combined with prednisolone.^8^

For systemic lupus erythematosus with cutaneous manifestations and nephritis, 1 mg/kg daily can be added to cyclophosphamide (0.05 to 1.00 g/m^2^ body surface per month for six months).^34^

### Dermatomyositis

In retrospective case series, it has been shown to be effective. It is considered equally effective when compared to methotrexate. If patients have an inadequate response to these medications, combined treatment offers good results in refractory disease.^35^ It is also recommended to prevent relapses for long periods (one to three years).^36^

### Other uses in dermatology

In an open-label pilot, uncontrolled study for moderate and severe alopecia areata a 2 mg/kg dose provided remarkable improvement.^37^ In a prospective study with 14 patients diagnosed with the universalis variant, recalcitrant to other systemic and topical therapies, 43% had a therapeutic response with a mean dose of 142 mg daily and a mean duration of therapy of ten months. Total regrowth was seen in 63% and 29% maintained response at 18 months of follow-up.^38^

One case with eosinophilic fasciitis and generalized morphea was treated with 200 mg/day for two months with subsequent tapering to 100 mg/day. Remission was maintained 18 months after treatment.^39^

In sarcoidosis, it has demonstrated improvement in some cases; however, compared with methotrexate, azathioprine exhibited a similar response but greater toxicity.^40^

Bullous pemphigoid and psoriasis can co-exist, and cases have been successfully managed with azathioprine (50–150 mg/day). It prevents reactivation of psoriasis when interrupting steroids.^41^ In conjunction with acitretin it is used for erythrodermic psoriasis and bullous pemphigoid.^42^

### Adverse effects/toxicity

#### Short term

##### Nausea

This is the most frequent dose dependent adverse effect. It arises at the beginning of treatment and improves even without altering the dose. To avoid it, doses are started at their lowest range; <2.5 mg/kg reduces abandonment rate by 20%.^5^ The dose can be split and taken with meals.^3^

##### Hypersensitivity to azathioprine

This idiosyncratic reaction is immunologically mediated. It presents within a few weeks of starting the medication. Identification is essential since it may be misinterpreted as a sign of infection. Patients may have fever, myalgia, arthralgia, nausea, hepatitis, interstitial nephritis, renal failure, and pneumonitis.^43^ Cutaneous signs include erythema nodosum, Sweet's syndrome, small vessel vasculitis, acute generalized pustulosis, and other non-specific dermatoses. Hypotension and shock may develop in severe cases.^44^

It is possibly underdiagnosed, and atopic eczema patients are the most likely to develop it. Cutaneous signs and symptoms resolve five days after interrupting the drug.^5^, ^44^

#### Medium term

##### Myelotoxicity

This is characterized by leukopenia, thrombocytopenia, anemia, and/or pancytopenia.^7^ Neutropenia is dose dependent. This serious side effect may happen more often than presumed. Approximately 19% of patients develop neutropenia.^5^

Clinical infection, pharyngeal ulceration, ecchymosis, and/or bleeding can lead to its identification. Monitoring is required if there is a decrease in platelet and white blood cell count within the normal lower limit. If lymphocytes decrease <0.50 k/uL, it is advisable to reduce the dose. If alterations in platelet count show <50 k/uL and/or neutrophils are <1.0 k/uL, a hematology consultation is recommended.^3^

##### Infections

There is increased susceptibility to infections due to lymphopenia even without neutropenia. Latent tuberculosis patients are at risk of reactivation when starting treatment.^5^

##### Hepatotoxicity

Reversible portal fibrosis and/or minimal cholestasis may develop.^7^ Mild changes in liver function tests without serious clinical implications are frequent. There are two types of hepatotoxicity for azathioprine: idiosyncratic acute liver injury, with hepatocellular disease (severe transaminase increase) or cholestasis (increase in alkaline phosphatase and bilirubin).^5^ These resolve by decreasing or withdrawing medication.^3^, ^5^ Serious hepatotoxicity is rare.^5^

#### Long term

There is a risk of cutaneous infections and hematological or cutaneous neoplasms.^7^ However, when used exclusively for dermatological conditions, this is unlikely since the dosage is lower and time of use is shorter.^1^

## Conclusion

Azathioprine is a useful medication in patients with complex dermatologic conditions and/or resistant to conventional treatments. It has been approved for diseases like lupus, dermatomyositis, and pemphigus vulgaris. It is essential for the dermatologist to adequately educate the patient who will receive the medication for its adverse effects. Physicians should be aware that these undesired effects improve and resolve when azathioprine is decreased or interrupted. It is always recommended to start at the lowest possible dose in order to improve tolerance and to avoid permanent discontinuation of a drug that can be extremely beneficial for the patient.

## Financial support

None declared.

## Authors’ contributions

Sonia Chavez-Alvarez: Statistical analysis; approval of the final version of the manuscript; design and planning of the study; drafting and editing of the manuscript; collection, analysis, and interpretation of data; critical review of the literature; critical review of the manuscript.

Maira Herz-Ruelas: Critical review of the literature.

Alejandra Villarreal-Martinez: Design and planning of the study; drafting and editing of the manuscript.

Jorge Ocampo-Candiani: Approval of the final version of the manuscript; effective participation in research orientation.

Rubicela Garza-Garza: Effective participation in research orientation; critical review of the manuscript.

Minerva Gomez-Flores: Design and planning of the study; intellectual participation in the propaedeutic and/or therapeutic conduct of the studied cases; critical review of the literature.

## Conflicts of interest

None declared.
